# Artificial optimization of bamboo *Ppmar2* transposase and host factors effects on *Ppmar2* transposition in yeast

**DOI:** 10.3389/fpls.2022.1004732

**Published:** 2022-10-20

**Authors:** Xiaohong Zhou, Jiamin Xie, Chao Xu, Xiuling Cao, Long-Hai Zou, Mingbing Zhou

**Affiliations:** State Key Laboratory of Subtropical Silviculture, Institute of Bamboo Research, Zhejiang A&F University, Hangzhou, China

**Keywords:** *mariner-like element (MLE)*, artificial optimization, transposase, host factor, bamboo, yeast

## Abstract

*Mariner-like elements* (*MLEs*) are promising tools for gene cloning, gene expression, and gene tagging. We have characterized two *MLE* transposons from moso bamboo, *Ppmar1* and *Ppmar2*. *Ppmar2*, is smaller in size and has higher natural activities, thus making it a more potential genomic tool compared to *Ppmar1*. Using a two-component system consisting of a transposase expression cassette and a non-autonomous transposon cotransformed in yeast, we investigated the transposition activity of *Ppmar2* and created hyperactive transposases. Five out of 19 amino acid mutations in *Ppmar2* outperformed the wild-type in terms of catalytic activities, especially with the S347R mutant having 6.7-fold higher transposition activity. Moreover, 36 yeast mutants with single-gene deletion were chosen to screen the effects of the host factors on *Ppmar2NA* transposition. Compared to the control strain (*his3Δ*), the mobility of *Ppmar2* was greatly increased in 9 mutants and dramatically decreased in 7 mutants. The transposition ability in the *efm1Δ* mutant was 15-fold higher than in the control, while it was lowered to 1/66 in the *rtt10Δ* mutant. Transcriptomic analysis exhibited that *EFM1* defection led to the significantly impaired *DDR2*, *HSP70* expression and dramatically boosted *JEN1* expression, whereas *RTT10* defection resulted in significantly suppressed expression of *UTP20*, *RPA190* and *RRP5*. Protein methylation, chromatin and RNA transcription may affect the *Ppmar2NA* transposition efficiency in yeast. Overall, the findings provided evidence for transposition regulation and offered an alternative genomic tool for moso bamboo and other plants.

## Introduction

Transposons are found in a wide variety of species and migrate across the genome. There are two types of transposons: DNA transposons and RNA transposons. *Mariner-like elements* (*MLEs*) belong to the DNA transposon family. *Mariner* transposon was first characterized in the *Drosophila mauritania* mutant with white eyes, and later homologous elements were discovered in abundance in plant and animal genomes (Hartl et al., 1997). *MLEs* are typically comprised of an Open Reading Frame (ORF) that encodes the transposase (Tpase), which locates between two paired Terminal Inverted Repeats (TIRs) and Target Site Duplications (TSDs) (Plasterk et al., 1999). Transposase has three well-defined domains. The N-terminal domain contains helix-turn-helix (HTH) motifs that recognize and bind the TIRs. The C-terminus contains a catalytic domain with a DDE/D triad (Yuan and Wessler, 2011). The two aspartic acid residues (D) and a glutamic acid (E) or another aspartic acid (D) are commonly located 34 or 39 amino acids apart in animal and plant transposases (DD34E/D, DD39D, respectively) (Hartl et al., 1997; Feschotte and Wessler, 2002). The Linker region, harboring a conserved WVPHEL motif, connects the HTH motif and catalytic domain (Liu and Chalmers, 2014).

The conservation of the transposase sequence has a significant impact on transposition efficiency. The three most well-studied *MLE*s to date are *Sleeping Beauty* (*SB*) from fish (Ivics et al., 1997), *Mos1* from *Drosophila mauritiana* (Bryan et al., 1990), as well as *Himar1* from *Haematobia irritans* (Robertson and Lampe, 1995). In *SB*, a single amino acid mutation yielded around 2-3-fold transposition efficiency, and 9 amino acid mutations (K14R, K33A, R115H, RKEN214-217DAVQ, M243H, T314N) by DNA shuffling resulted in a hyperactive mutant SB100X with a 100-fold increase in transposition activity (Mátés et al., 2009). Germon et al. (2009) used systematic single amino acid substitutions to create two hyperactive *Mos1* mutants (FETY and FET) that were 60- and 800-fold more active than the wild-type *Mos1* version. The Linker region is also important for transposition efficiency. Almost all single mutations of the WVPHEL motif in *Himar1* and *Hsmar1* transposase led to highly active transposase variants, but which easily produce non-productive DNA double-strand breaks that can induce DNA damage and mutations (Butler et al., 2006; Lampe, 2010; Liu and Chalmers, 2014).

Besides the sequences of transposases, DNA methylation, chromatin status, over-production inhibition (OPI) and host proteins may influence the transposition frequency. It was shown that the methylated *SB* was at least 100-fold more active than the unmethylated version. CpG methylation of the *SB* region and heterochromatin formation facilitated the transposition reaction (Yusa et al., 2004). The MLE transposase activity was also inhibited by OPI (Lohe and Hartl, 1996). Overproduction of wild-type transposase enhanced the attachment of the transposase dimer and competition for free transposon ends, ultimately lowering the transposase activity. OPI started to occur when the number of transposase dimers was superior to the number of available TIRs (Claeys et al., 2013). High Mobility Group B1 (HMGB1) was a host-encoded cofactor of *SB* transposition and was involved in the formation of transposase-transposon complexes. Transposition of *SB* was severely suppressed in the HMGB1-deficient mouse cells (Zayed et al., 2003). In yeast (*Saccharomyces cerevisiae*), more than 200 host factors have been found to be associated with *Ty1* and *Ty3* retrotransposon (Irwin et al., 2005; Curcio et al., 2015). These factors were hypothesized of being involved in chromatin and transcript elongation, translation and cytoplasmic RNA processing, vesicular trafficking, nuclear transport, and DNA maintenance.

Two full-length *MLEs* named *Ppmar1* and *Ppmar2* were previously identified from the genome of moso bamboo (*Phyllostachys edulis*) (Zhou et al., 2015). *Ppmar1* and *Ppmar2* were shown to be transposable in *Arabidopsis thaliana* and yeast (Zhou et al., 2016; Zhou et al., 2017; Ramakrishnan et al., 2019a; Ramakrishnan et al., 2019b). Site-directed mutation boosted the activity of the *Ppmar1* mutant (*S171A*) by more than 10-fold (Zhou et al., 2017). *Ppmar2* is smaller in size (Zhou et al., 2016) and has a higher natural activity (Zhou et al., 2017), thus making it a more potential genomic tool compared to *Ppmar1*. Moso bamboo has a very lengthy vegetative growth cycle (~60 years), and hence rarely reproduces sexually, but reproduces *via* rhizomes during the vegetative period (Janzen, 1976; Watanabe et al., 1982). Breeding of moso bamboo *via* hybridization is extremely difficult due to its occasional sexual reproduction. So, a bamboo mutant library *via* an efficient transformation system will be a preference for future breeding. Currently, the most used genomic tools are T-DNA insertion and gene targeting (e.g., CRISPR/Cas). Insertional mutagenesis based on active transposons may be a promising tool to manipulate the genome of moso bamboo.

In the present study, we focused on the *Ppmar2* transposon to develop hyperactive *Ppmar2* mutants by rational mutagenesis. The host factors in yeast which regulated *Ppmar2* transposition were also screened. The hyperactive *Ppmar2* transposon system reported in this study with its outstanding features including its compact size and non-linked insertion sites could provide an alternative genomic tool for moso bamboo and other plants.

## Methods

### Construction of the *Ppmar2* transposition system in yeast

The *Ppmar2* transposition system was constructed using the transposon donor vector, pWL89A, and the transposase expression vector, pAG415gal-ccdB, as described by Ramakrishnan et al. (2019a; 2019b). *Ppmar2* non-autonomous transposon (*Ppmar2NA*) was cloned by amplifying its 5’ and 3’ TIRs as well as the adjacent sequence, followed by an overlap PCR. *Ppmar2NA* was inserted into the *Xho I* site at the 5’ untranslated region (UTR) of the *Ade2* gene in the vector pWL89A possessing two selectable markers, *Ura3* and *Ade2*. The transposon donor vector was named pWL89A-Ppmar2NA. The *Ppmar2* transposase sequences were amplified by adding *Not I* and *EcoR V* sites at both ends to fit the pAG415gal-ccdB vector. Both restriction enzymes cut the pAG415gal-ccdB vector. The transposase fragment and the backbone of pAG415gal-ccdB were then ligated by T4 DNA ligase to obtain the recombined vectors pAG415gal-transposase with *Leu2* selectable marker. The transposase was promoted to be expressed under the *gal* promoter.

### Yeast transposition assay

The two prepared plasmids (pWL89a-Ppmar2NA and pAG415gal-transposase) were co-transformed into *S. cerevisiae* strain DG2523. Transformed yeast strains were grown on medium lacking Leucine and uracil (SD-his-ura) but with 2% galactose at 30°C in the dark for 3 days, followed by the suspension of single colonies in 150 μl water and plating onto medium lacking adenine, Leucine, and uracil (SD-ade-his-ura) with 2% galactose as the carbon sources. The plates were incubated at 30°C in the dark for about 3 days to allow the growth of ADE2 revertant colonies.

### Transposition footprints analysis

The fragments covered *Ppmar2NA* excision spots on pWL89a were amplified using primers of yeast T-5 (5’-CAC CCC AGG CTT TAC ACT TTA TG-3’) and T-3 (5’-GTT GCT TAT TTG TTT GGC AGG AG-3’). PCR products were cloned into the pUC18-T vector for sequencing.

### Insertion sites and insertion bias analysis

Genomic DNA was extracted from the single ADE2 revertant colonies and then was sheared into 500-bp fragments using Covaris E220 ultrasonicator (Covaris, UK). The libraries were normalized, pooled, and sequenced *via* Illumina high-throughput sequencing platform NovaSeq 6000 (2×250 bp paired-end run). Low-quality sequences were filtered by Sickle (v1.33) with Q30 and 125bp minimal length (Joshi and Fass, 2011). Filtered reads were aligned to the *Ppmar2NA* sequence through the local blast. Subsequently, the reads containing the *Ppmar2NA* sequence and adjacent sequence were aligned to the *S. cerevisiae* reference genome (https://yeastgenome.org/) to identify the insertion sites. The 20bp upstream and downstream of the insertion sites were aligned to verify the nucleotide distribution characteristics of the *Ppmar2NA* insertion sites.

### Site-directed mutagenesis of *Ppmar2* transposase

To identify *Ppmar2* non-conserved transposase sites, we downloaded 22 *MLE* transposase sequences from GenBank and aligned them with the *Ppmar2* transposase using MEGA11.0.10 (Tamura et al., 2021). Mutagenesis was performed with the QuikChange Lightning Site-Directed Mutagenesis Kit using the primers listed in **Supplementary Table S1**. The selected sites of the *Ppmar2* transposase were mutated into corresponding amino acids (**Supplementary Table S2**). The mutated *Ppmar2* transposases then replaced the template on pAG415gal-transposase to examine the transposition activities. All plasmids were sequenced to confirm the presence of the targeted mutation. Homologous hyperactive mutation sites in the *Mos1* and *Himar1* transposase were mutated into the corresponding amino acid in the *Ppmar2* transposase.

### Transposition frequency of *Ppmar2* mutants

The transposition frequencies of *Ppmar2* mutants were evaluated by ADE2 revertant frequencies. Each galactose-induced colony was suspended in 50 μl of water and plated on media without adenine. The cell suspension was equally diluted to 1×10^-5^ volume and was plated on SD media lacking adenine, Leucine and uracil (SD-ade-his-ura) to obtain the total number of galactose-induced colonies. The assay of each mutant was performed with six biological replicates.

### Transposition frequency analysis of *Ppmar2* in yeast mutants

To examine the host factors’ effects on *Ppmar2* transposition in yeast, 36 mutants with single-gene deletions were selected (**Table S3**) which were kindly provided by Charles Boone (Toronto University, Toronto, in Canada), and *His3Δ* was used as the control strain. These genes are involved in methylation, DNA replication, transcription, translation, as well as the regulation of retrotransposon (*Ty1* and *Ty3*) (Irwin et al., 2005; Curcio et al., 2015).

### Gene expression *via* RNA-seq

The *efm1Δ*, *rtt10Δ* mutants and the wild type yeast *His3Δ* were cotransformed with pWL89a-Ppmar2NA and pAG415gal-transposase plasmids with three colonies for each genotype. The mRNA of the single ADE2 revertant colonies was extracted, when strains grew in YPD liquid medium with the OD600 of 0.5, and was synthesized into cDNA strands with dNTPs and DNA polymerase I. The final cDNA library was obtained through the AMPure XP system and was sequenced on an Illumina Hiseq 2000 platform (Tianjin Nuohe Zhiyuan Bioinformatics Co., Ltd.) to generate 125 bp/150 bp paired-end reads. The adapter, ploy-N and low-quality reads from raw data were removed using Illumina PIPELINE software. Index of the yeast genome has been built using STAR (v2.5.1b) and paired-end clean reads were aligned to the yeast reference genome (https://yeastgenome.org/) with TopHat v2.0.12. The reads mapped to each gene were counted by HTSeq v0.6.0 and were normalized to fragments per kilobase of exon per million fragments mapped (FPKM) (Trapnell et al., 2010). The method of Benjamini and Hochberg was used to adjust the *P*-value to control the error detection rate (Haynes, 2013). In this experiment, the correction of *p* < 0.05, with | log2foldchange | > 1 was used for screening differentially expressed genes (DEGs).

### Quantitative real-time PCR analysis

Eight DEGs were selected for qRT-PCR analysis. One μg total RNA of each sample was reverse-transcribed using PrimeScript™ RT reagent Kit with gDNA Eraser. The qRT-PCR was performed using the SYBR^®^ Premix Ex Taq™ II (Tli RNaseH Plus) on the iQ™5 Multi-channel Real-time PCR Detection System (Bio-Rad). The relative abundance of each gene expression was calculated from the 2-ΔΔCt values between the target gene and *ALG9 (Protein amino acid glycosylase 9)* (Teste et al., 2009). The results were analyzed by Bio-Rad CFX Manager 3.1 software. Each reaction was performed at least three times.

## Results

### Verification of the *Ppmar2* transposition potential in yeast

To determine whether *Ppmar2* can transpose in the yeast genome, we performed transposition assays in yeast utilizing the methods described previously (Ramakrishnan et al., 2019a; Ramakrishnan et al., 2019b). The assays used two constructs, one transposase expression vector and one transposon donor vector. In the transposase expression plasmid, the *Ppmar2* transposase coding sequence was fused to the inducible *GAL1* promoter, and *Leu2* served as the selectable marker. In the transposon donor plasmid, the non-autonomous *Ppmar2NA* element was inserted in the 5’UTR of the *ADE2* reporter gene, and *Ura3* served as the selectable marker. Transformants containing both plasmids were selected on a medium with 2% galactose but lacking Leucine and uracil. Colonies of the double transformants were picked and regrown on agar plates without adenine for the selection of *ADE2* revertants, which represented the excision of the *Ppmar2NA*. Notably, *ADE2* revertant colonies were obtained in the presence of the *Ppmar2* transposase, but none when the control plasmid, pAG415-ccdB, was used.

**Figure 1:**
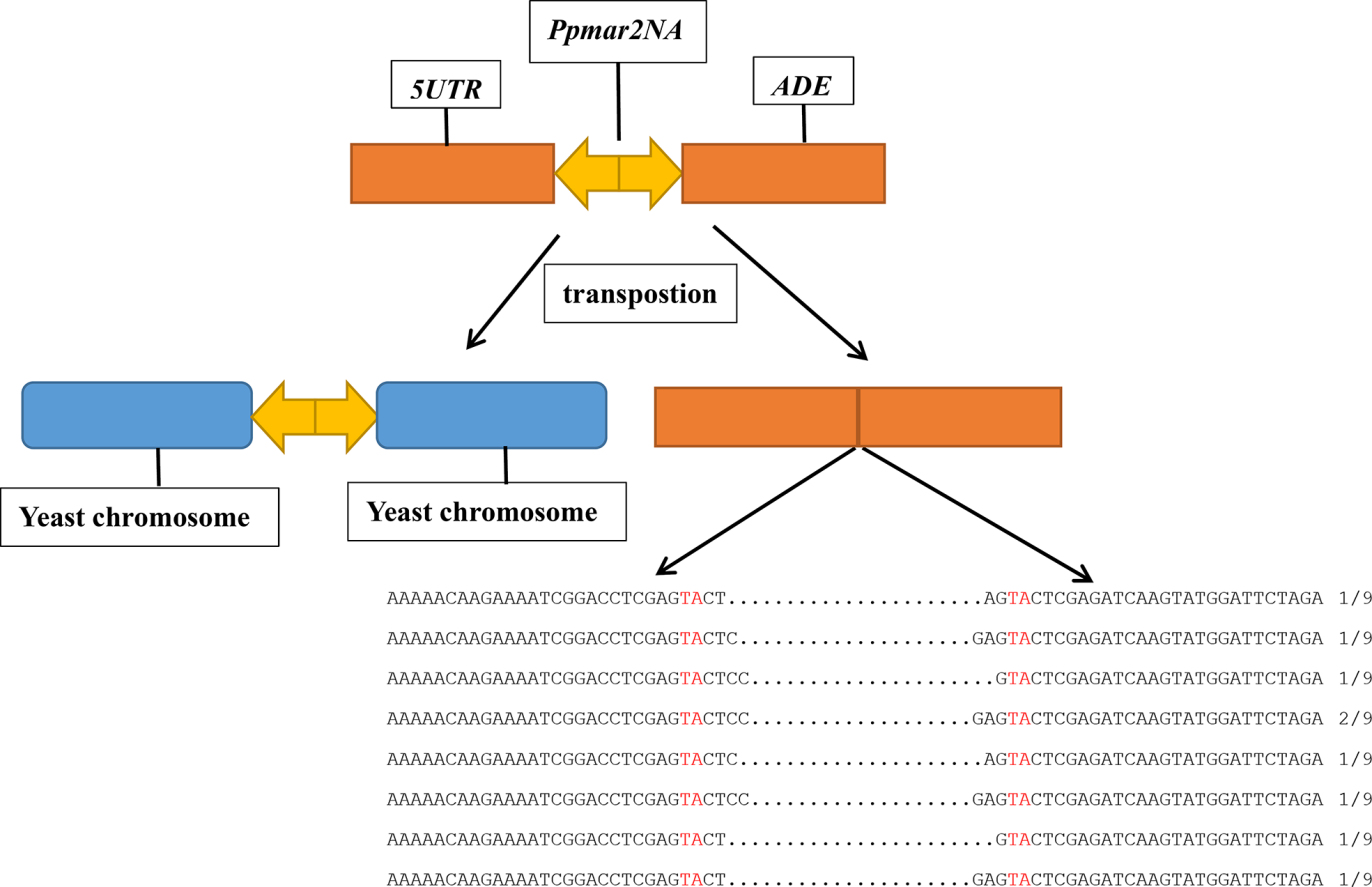
Sequence alignment of *Ppmar2NA* at excision spots on pWL89a. Sequences of *Ppmar2NA* at excision spots were amplified using primers yeast T-5 and T-3. The TA-target sites of TSDs are marked in red, and the interval between the red TA represents the footprints after cleavages.

Plasmid DNA was prepared from the independent *ADE2* revertants, and the excision products of *Ppmar2NA* in the *ADE2* 5’UTR region were PCR amplified using primers of yeast T-5 and T-3. Sequencing results revealed diverse footprints of excision were generated by *Ppmar2NA* in the donor plasmid. Between two *Ppmar2NA* TIRs, one to four bases were retained (**Figure 1**).

### Reinsertion preferences of the excised *Ppmar2NA*

**Figure 2:**
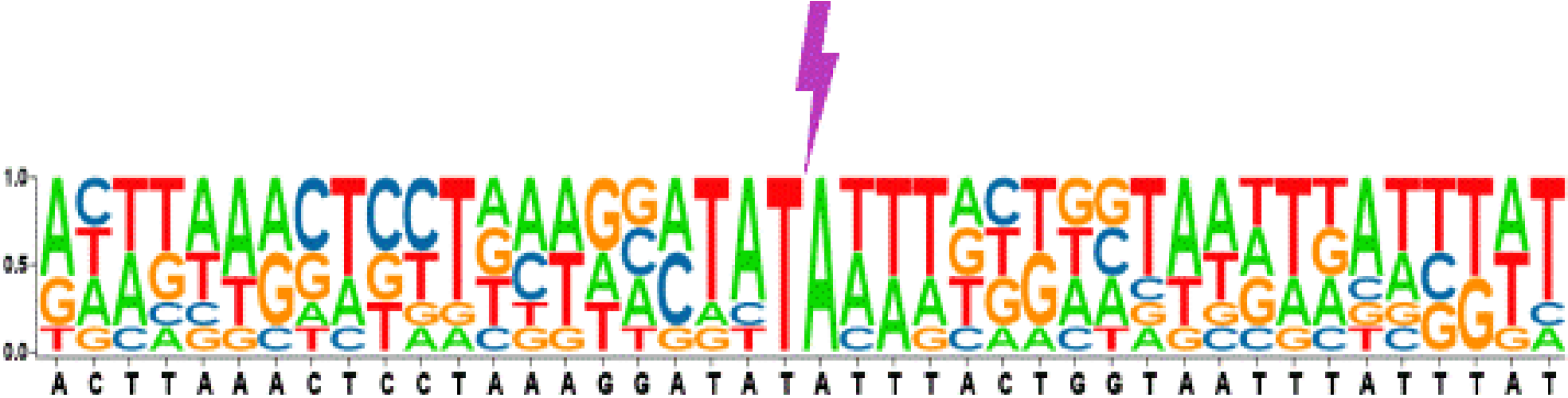
Reinsertion preference analysis of excised *Ppmar2NA*. The pictogram shows the relative nucleotide frequencies of the 7 detected insertion events in the yeast genome. On either side of the reinserted *Ppmar2NA* elements, 20 bases flank the conserved TA insertion spot. The nucleotide A is shown in green, C in red, G in blue, and T in yellow. The lightning symbol indicates the *Ppmar2NA* reinsertion site.

To follow the fate of the excised *Ppmar2NA* and localize the reinsertion sites in the yeast genome, genomic DNA was extracted from the independent *ADE2* revertants, and sequenced (with 30 times coverage). Following alignment, we identified 7 genetic integration events on 6 yeast chromosomes. All insertions occurred within ~500 bp of the coding regions, with one insertion occurring within the gene (**Table 1**). As expected, *Ppmar2NA* in all detected events was inserted into a TA dinucleotide where the AT content of 50 bp sequences nearby the insertion sites was more than 60% (**Figure 2**). To further validate the *Ppmar2NA* insertion bias, we analysed the 20 bp sequences flanking the TA on both sides. Alignment of the 7 integration sites revealed that the excised *Ppmar2NA* was preferentially inserted into AT-rich regions (**Figure 2** and **Table 1**).

**Table 1:** Details of *Ppmar2NA* reinsertion sites.

No. of chromosome	Insert site	AT content of 50 bp sequences around the insertion sites	Distance from the nearest gene
I	138841	68%	495 bp upstream from *ERP2* gene
IV	26011	79%	33 bp downstream from *LRG1* gene
IV	524715	62%	20 bp upstream from *EHD3*
IX	33347	65%	380 bp downstream from *YIL165C* gene
X	585548	66%	110 bp upstream from *YJR085C* gene
XIII	302740	63%	254 bp upstream from *ERG5* gene
XVI	541299	61%	Coding region of *CHL1* gene

### Targeted mutagenesis of the *Ppmar2* transposase to enhance *Ppmar2NA* transposition in yeast

**Figure 3:**
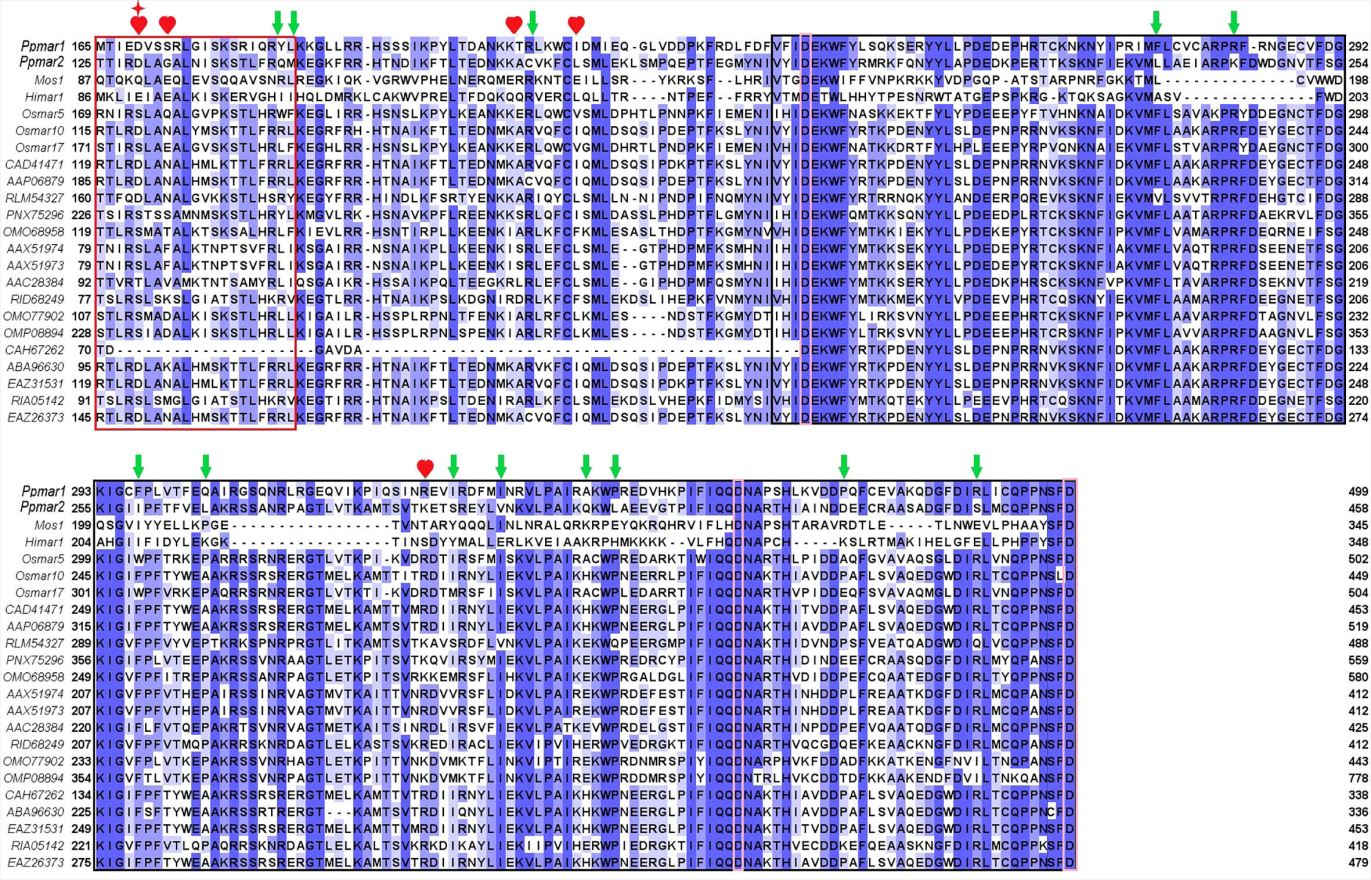
Homology alignment of 22 *MLE* transposases. The red box is the HTH domain, the black box is the DDD domain, and the Linker area is in the middle. Three aspartic acid residues (D) in the DDD domain were highlighted with pink boxes. *Mos1* from *D. mauritiana* (Bryan et al., 1990), *Himar1* from *H. irritans* (Robertson and Lampe, 1995), *Ppmar1* from moso bamboo and three *MLE* transposases (*Osmar5, Osmar10, Osmar17*) from rice (Yang et al., 2009). Other entries are named according to the first letter of the genus name and the species name, followed by the GenBank accession number. The 12 non-conserved sites (marked by green arrows) of the *Ppmar2* transposase were mutated into corresponding conserved amino acids. The 6 homologous hyperactive mutation sites in the *Mos1* and *Himar1* transposase were mutated into the corresponding amino acid in the *Ppmar2* transposase (marked by a red heart shape). The amino acid at the 129^th^ position was mutated into two different amino acids (marked by a red star).

To improve the transposition activity of *Ppmar2*, the transposase was modified following the strategy as described (Zhou et al., 2017). We selected 22 *MLE* transposase sequences from animals and plants, including *Mos1* from *D. mauritiana* (Bryan et al., 1990), *Himar1* from *H. irritans* (Robertson and Lampe, 1995), *Ppmar1* from Moso bamboo, and three *MLE* transposases from rice (Yang et al., 2006; Yang et al., 2009) (**Figure 3**). Other sequences were filtered in the NCBI database by blast using the *Ppmar2* transposase sequence as a query, which showed high homology with the *Ppmar2* transposase. We examined amino acid residues that were partially conserved in *MLEs* but not in the *Ppmar2* transposase after alignment. Thirteen such sites scattered across the three conserved domains of the *Ppmar1* transposase (the DNA binding domain, the Linker domain, and the catalytic domain) were identified (**Figure 3**). Moreover, we selected six homology sites in the *Ppmar2* transposase that corresponded to the hyperactive mutation sites in the *Mos1* and *Himar1* transposase (D129A, D129R, G132A, A168R, L174K and K289A) (**Figure 3**) (Butler et al., 2006; Germon et al., 2009). The 18 candidate sites, including four in the HTH binding domain, three in the Linker region, and eleven in the catalytic domain, were systematically modified one by one, and the transposase variants were evaluated in the yeast excision assay (**Table S2**).

**Figure 4:**
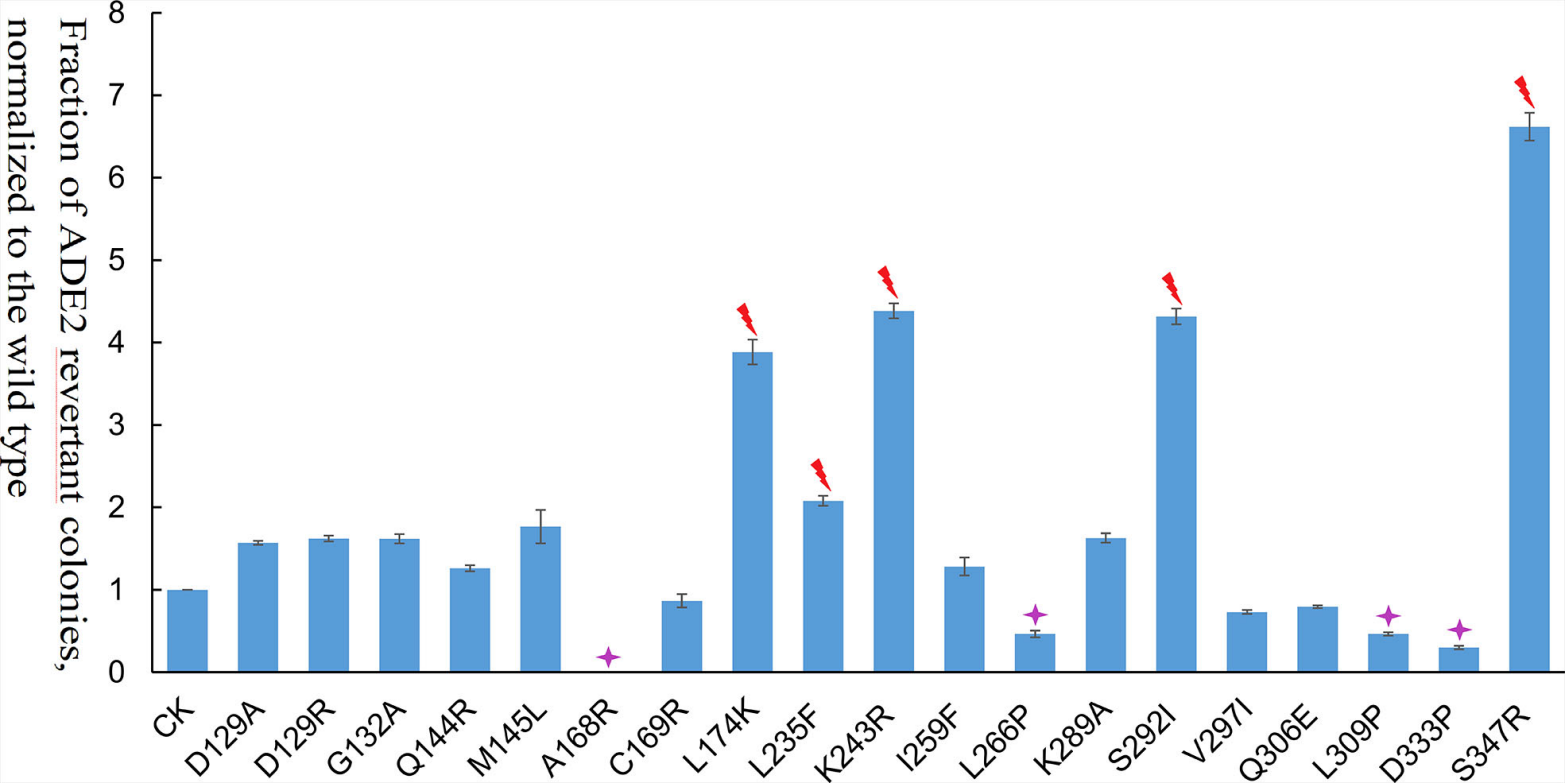
Transposition frequencies catalyzed by *Ppmar2* transposase mutants (CK is the wild type *Ppmar2* transposase). The mean excision frequency of the 19 *Ppmar2* mutants was normalized by that of the wild type. Y-axis represents the fraction of ADE2 revertant colonies, normalized to the wild type. Each excision assay was conducted with six biological replicates. The transposase mutations were marked by red heart shapes which dramatically enhanced *Ppmar2* transposase catalytic activity by more than 2-fold. The transposase mutations were marked by pink star shapes which dramatically reduced *Ppmar2* transposase catalytic activity to less than 1/2-fold.

Among the 19 transposase mutations (two mutations at the 129th amino acid), 5 of them (L235F, K243R, S347R, S292I, and L174K) dramatically enhanced the *Ppmar2* excision activity by more than 2-fold (**Figure 4**). Four amino acid substitutions, including L266P, L309P, D333P, and A168R, suppressed the transposition. The remaining 10 amino acid substitutions had no significant effects (**Figure 4**). The S347R mutant exhibited the highest transposition activity, which was 6.7-fold that of the wild type (**Figure 4**).

### Host factors’ effects on *Ppmar2* transposition in yeast

**Figure 5:**
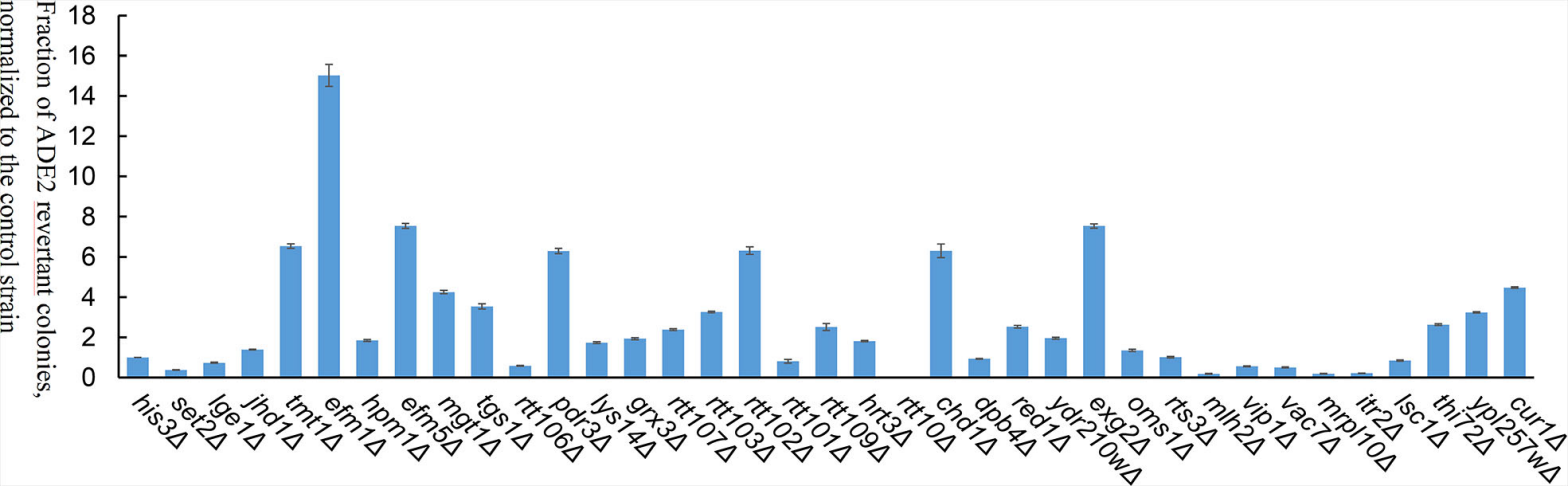
Transposition efficiency of *Ppmar2NA* in 36 yeast single-gene mutants. The mean excision frequency of *Ppmar2NA* in the 36 yeast mutants was normalized to that of the control strain (*His3Δ*). Y-axis represents the fraction of ADE2 revertant colonies, normalized to the control strain. Each excision assay was conducted with six biological replicates.

To investigate the effects of host factors on transposition efficiency, 36 yeast mutants with single-gene deletions were screened (**Table S2**). Of the 36 genes, 8 genes were involved in methylation, 7 genes in DNA replication, transcription, and translation, and 21 genes were host factors of retrotransposons (*Ty1* and *Ty3*) (Irwin et al., 2005; Curcio et al., 2015). The 36 yeast mutants were double transformed with pWL89a-Ppmar2NA and pAG415gal-transposase. The positive colony assay in each yeast mutant revealed that the transposition frequency was significantly higher in 9 yeast mutants (*tmt1Δ, efm1Δ, efm5Δ, mgt1Δ, pdr3Δ, rtt102Δ, chd1Δ, exg2Δ*, and *cur1Δ*) than in the control strain (*His3Δ*), but significantly lower in 7 yeast mutants (*set2Δ, rtt106Δ, rtt10Δ, mlh2Δ, vac7Δ, mrpl10Δ*, and *itr2Δ*), with no obvious differences in the other 20 yeast mutants (**Figure 5**). *Ppmar2NA* showed the most active transposition in the *efm1Δ* mutant, which was 15-fold higher than in *His3Δ*. *Ppmar2NA* had the lowest transposition frequency in the *rtt10Δ* mutant, which was only 1/66 that in the *His3Δ* strain.

### DEGs in the mutants with dramatically distinct transposition ability *Ppmar2NA*

**Figure 6:**
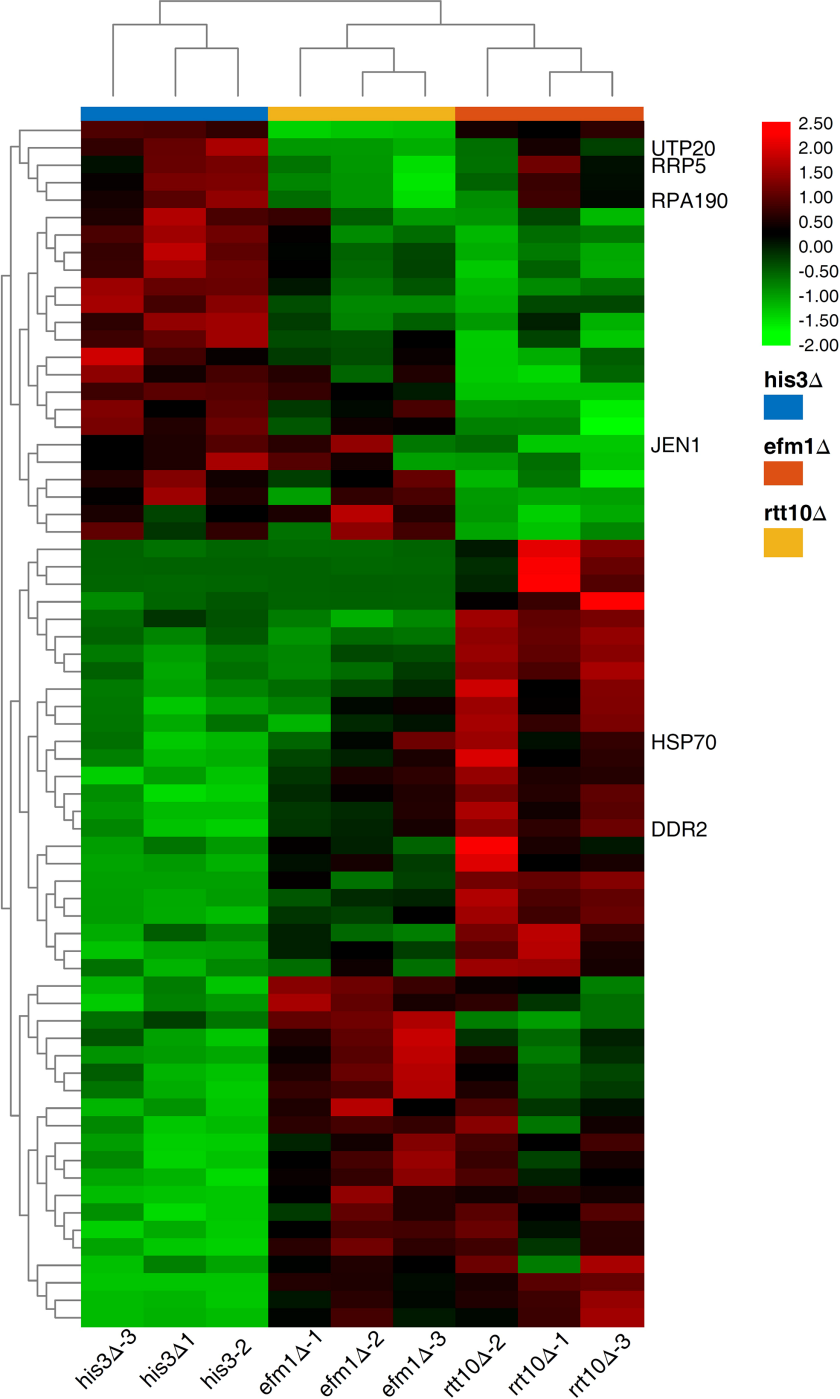
Heat map of DEGs expression in *HisΔ3, efm1Δ, rtt10Δ* strains. The expression patterns of *DDR2, HSP70, JEN1, UTP20, RPA190* and *RRP5* genes were marked. The color scale indicates the relative expression level.

To explore the host factors’ effects and investigate the DEGs involved in the transposition regulation, we performed the transcriptome analyses for the strains with distinct transposition competence *Ppmar2NA* (*efm1Δ* vs. *His3Δ* and *rtt10Δ* vs. *His3Δ*). Sixty-seven DEGs were validated with a *P*-value lower than 0.0001 (**Figure 6**) and enriched *via* GO and KEGG. These DEGs were related to ribosome biogenesis, RNA modification and DNA modifications which might be involved in the regulation the *Ppmar2* transposition (**Figures 1S**, **2S**). In the mutant *efm1Δ* with highly active *Ppmar2NA*, three genes linked with DNA repair and RNA transcription (*DNA Damage Responsive 2* (*DDR2*), *Heat Shock Protein 70* (*HSP70*) and *Monocarboxylate/proton symporter of the plasma membrane* (*JEN1*)), might be likely related to regulation of the *Ppmar2NA* transposition. *DDR2* and *HSP70* were highly expressed in the *efm1Δ* strain, while the expression of *JEN1* was repressed. In the *rtt10Δ* null mutant, three genes (*U3 snoRNA-associated protein 20* (*UTP20*)*, DNA-directed RNA polymerase I subunit* (*RPA190*)*, U3 small nucleolar RNA-associated protein* (*RRP5*)), involved in ribosome assembly and RNA polymerase synthesis, were markedly downregulated.

qRT-PCR was performed to validate the RNA-seq data by checking the expression levels of *Elongation Factor Methyltransferase (EFM1)*, Regulator of Ty1 Transposition (*RTT10*)*, DDR2, HSP70, JEN1, UTP20, RPA190* and *RRP5*. No expression of *EFM1* was detected in *efm1Δ*, and a low-level expression of *RTT10* in *rtt10Δ*, as predicted. The other gene expression patterns displayed *via* qRT-PCR were completely consistent with the RNA-seq data-derived gene expression pattern (**Supplementary Figure 3**).

## Discussion

Yeast has been widely used as a heterologous host to evaluate the transposition activity of plant DNA transposons, such as *Osmar5* and *Stowaways MITE* in rice (Yang et al., 2006; Yang et al., 2009), and *Ac/Ds* in maize (Lazarow et al., 2012). We have identified two *MLE* transposons in moso bamboo, *Ppmar1* and *Ppmar2*, which both could jump in *Arabidopsis thaliana* and yeast genome (Zhou et al., 2016; Zhou et al., 2017; Ramakrishnan et al., 2019a; Ramakrishnan et al., 2019b). *Ppmar2* has a relatively smaller size and naturally higher activity than *Ppmar1*, thus making it a more potential genomic tool. To improve the transposition efficiency and develop an alternative gene-tagging tool, we create a hyperactive *Ppmar2 via* mutagenesis and screen the host factors influencing transposition of *Ppmar2* in yeast.

### *Ppmar2NA*’s transposition footprints and insertion preferences resembled the other *MLEs*

Using a two-component system consisting of a transposase expression cassette and a non-autonomous transposon cotransformed in yeast, we examined the transposition activity of *Ppmar2NA*. The footprints of *Ppmar2NA* after excision revealed that *Ppmar2NA* was preferentially cut between the two TIRs, leaving 1-4 staggered nucleotides (**Figure 1**). Similarly, the *Osmar5* transposase generated a staggered cut with one to four nucleotides at both ends of the transposon element (Yang et al., 2006), whereas the animal transposases *Mos1*, *Sleeping Beauty*, and *Frog Prince* were cleaved but left no more than three nucleotides (Luo et al., 1998; Dawson and Finnegan, 2003; Miskey et al., 2003). On the other hand, *Ppmar2NA* tended to insert into the TA-rich regions and generated AT TSDs (**Figure 2**). In short, as a member of the *MLEs* family transposons, *Ppmar2NA* shared a similar cleavage footprint and insertion preference.

### Some amino acids in key sites are important for the catalytic activity of *Ppmar2* transposase

Eighteen non-conversed amino acids in *Ppmar2* transposase, including 4 in the HTH binding domain, 3 in the linker junction region, and 11 in the DDD catalytic domain, were selected for mutagenesis to improve the transposition activity (**Figure 3**). Another 6 homology sites in *Ppmar2* corresponding to hyperactive mutation sites in *Mos1* and *Himar1* were also chosen (D129A, D129R, G132A, A168R, L174K and K289A) (Butler et al., 2006; Germon et al., 2009). The catalytic activities in five of the six mutants were improved, which was consistent with the mutation effect in *Mos1* and *Himar1* except for A168R (**Figure 4**) (Butler et al., 2006; Germon et al., 2009). Strangely, the five hyperactive mutation sites are not conserved in the sequences of *Ppmar2* transposase (**Figure 4**).

The linker region connects the HTH motif and catalytic domain, which are not conserved as WVPHEL motif in *Ppmar2* transposase. Three mutations (A168R, C169R, and L174K) located in the Linker region of the *Ppmar2* transposase, which regulated the spatial structure of the transposase, exhibited divergent transposition actives (Butler et al., 2006; Liu and Chalmers, 2014). The catalytic activity of the C169R mutation did not change significantly, but the A168R mutant totally lost the catalytic activity. The L174K transposase mutant, on the other hand, showed considerably higher catalytic activity than the wild type (**Figure 4**). The catalytic domain featured by a conserved DDE/D motif played a vital role in transposition activity (Yang et al., 2006). It has been reported that substitution of the DDD to the DDE in the *Tc1* family abolished the transposase activity (Lohe et al., 1997). The transposition efficiency of *Ppmar2* transposase varied among the targeted mutation sites (L235F, K243R, I259F, L266P, K289A, S292I, V297I, Q306E, L309P, D333P, and S347R). The L235F, K243R, S347R and S292I mutation greatly boosted the transposition. Especially, the S347R mutation resulted in a 6.7-fold higher transposition frequency (**Figure 4**). The hyperactive *Ppmar2* system would be used in plant genomic engineering as an alternative transposon-based technique.

It should be noted that the high catalytic activity of transposase mutations may not directly lead to high-frequency transposition due to OPI. Construction of transposase mutations which are usually less sensitive to OPI, e.g. low TIR binding affinity, and low stability of the transposase dimers will contribute to the high transposition frequency (Liu and Chalmers, 2014; Tellier and Chalmers, 2020). In the following, mutations that change in *Ppmar2* transposition kinetics that shift the OPI equilibrium will be considered.

### Host factors, including *EFM1* and *RTT10* genes, significantly affected the transposition frequency of *Ppmar2NA* in yeast

The yeast single-gene deletion mutant library was effectively used to investigate the impact of host factors on the activity of retrotransposons *Ty1* and *Ty3.* More than 200 host factors, including those involved in chromatin and transcript elongation, translation and cytoplasmic RNA processing, vesicular trafficking, nuclear transport, and DNA maintenance, regulated the transposon activity of yeast *Ty1* (Curcio et al., 2015) and *Ty3* (Irwin et al., 2005). The 36 related yeast mutants with single gene deletions were selected in this study. The transposition efficiencies of *Ppmar2NA* in 9 yeast mutants (*tmt1Δ, efm1Δ, efm5Δ, mgt1Δ, chd1Δ, pdr3Δ, cur1Δ, exg2Δ*, and *rtt102Δ*) were 2-fold more active than that in the control yeast. Notably, in the *efm1Δ* mutant, the transposition efficiency increased by 15-fold. Among the 9 genes, four are related to protein methylation, one to DNA methylation, one to chromatin organization, and the remaining 4 were host factors of retrotransposon. In another 5 yeast mutants (*set2Δ, rtt10Δ, mlh2Δ, mrpl10Δ*, and *itr2Δ*), the transposition efficiency of *Ppmar2NA* was at least 1/2 less active than in the control strain *His3Δ*, especially in *rtt10Δ* mutant, where the transposition efficiency decreased by more than 65-fold. One of the 5 genes was associated with histone methylation, whereas the other 4 genes were host factors of retrotransposon. These findings indicated that DNA transposon and RNA transposon may share common host factors (Irwin et al., 2005; Curcio et al., 2015).

In yeast, *Elongation Factor Methyltransferase (EFM1)*, which encoded lysine methyltransferase SET8, may characteristically methylate H4K20 (Zhang and Bruice, 2008) and function in protein modification, chromosome-protein binding regulation, and gene transcription and translation. Studies have shown that *EFM1* methylates H4K20 into three proteins H4K20me1, H4K20me2 and H4K20me3 (Yang and Mizzen, 2009). H4K20me1 could further promote chromatin condensation with the help of Condensin II (Weirich et al., 2015). Also, H4K20me1 and H4K20me2 inhibited gene transcription and chromosomal shrinkage by binding to the Lethal (3) Malignant Brain Tumor-Like Protein 1 (L3MBTL1) (Li et al., 2007). H4K20me3 was generally regarded as a marker of transcriptional inhibition in heterochromatin (Schotta et al., 2004). H4K20 was not methylated when the *EFM1* gene was knocked out, which might be related to the high transposition frequency of the *Ppmar2* in the *efm1Δ* mutant yeast.

The DEGs identified with the comparison of *efm1Δ* and *His3Δ* revealed that the *efm1Δ* cell conditions were changed significantly. The *DDR2* gene encoded a stress protein involved in DNA repair (Kobayashi et al., 1996). The expression of this gene was dramatically elevated in the *efm1Δ* mutant, suggesting that it may efficiently repair the damaged DNA. The HSP70, which encoded heat shock factor (Abrams et al., 2014), was also obviously upregulated in the *efm1Δ* mutant. Jardim et al. (2015) found evidence that *mos1* may be co-activated with *HSP70* genes. *JEN1* mediated high-affinity uptake of carbon sources lactate, pyruvate, acetate, and micronutrient selenite, *JEN1* expression and localization are tightly regulated, with transcription repression, mRNA degradation, and protein endocytosis and degradation all occurring in the presence of glucose (Haurie et al., 2001).

The *RTT10* gene, which encoded a member of the WD40 protein family, was a retrotransposition-related gene. *Ty1* was more active in the *rtt10Δ* mutant than in the *His3Δ* strain, and the *RTT10* gene might prevent *Ty1* RNA from being reversely transcribed into cDNA (Scholes et al., 2001). Moreover, *RTT10* contributed to the synthesis of transcription factor *TFIID*, regulating the ribosome small subunit rRNA synthesis (Dragon et al., 2002). Three DEGs (*UTP20*, *RPA190* and *RRP5*) in the *rtt10Δ* mutant might be related to the lowered transposition activity. The UTP20 protein was a component of the small-subunit (SSU) processome, which modulated the 18S rRNA synthesis (Gallagher et al., 2004). The expression level of this gene in the *rtt10Δ* mutant was significantly lowered. RRP5 was another component of the SSU processome and 90S preribosome related to the synthesis of 18S and 5.8S rRNAs (Venema and Tollervey, 1996). The expression level was much lower in the *rtt10Δ* mutant than in *His3Δ*. *RPA190*, encoding RNA polymerase A (Mémet et al., 1988), was also downregulated in the *rtt10Δ* mutant. The discovery of host factors associated with *Ppmar2* mobility lays an important foundation for understanding the mechanism of *Ppmar2* transposition in the heterologous host.

In the present study, we created hyperactive *Ppmar2* transposons *via* site-direct mutagenesis and screened the heterologous host factors that influenced the transposition activity. *Ppmar2* was proved to efficient to able to excision and reinserted into the TA-rich region, which was the same as the other *MLEs* in plants. *EFM1* and *RTT10* related to protein methylation, chromatin and RNA transcription greatly impact the heterologous transposition efficiency of *Ppmar2* in yeast. The *Ppmar2* transposon system is a promising tool for insertion mutagenesis in moso bamboo and might be used as an alternative to the existing transposon tagging systems in the other plants.

## Data availability statement

The datasets presented in this study can be found in online repositories. The names of the repository/repositories and accession number(s) can be found in the article/**Supplementary Material**.

## Author contributions

MZ designed the experiments; XZ and JX performed the research; CX, XC and L-HZ participated in the research; MZ and XZ wrote the manuscript. All authors have read and approved the manuscript.

## Funding

This work was funded by grants from the Zhejiang Provincial Natural Science Foundation of China (No. LZ19C160001 and LQ21C160003), the National Natural Science Foundation of China (No. 31870656, 31470615 and 32001326), the Talent Research Foundation of Zhejiang A&F University (No. 2019FR055) and Independent research project of state key laboratory of subtropical silviculture (ZY20200301).

## Acknowledgments

We would like to extend our sincere gratitude and appreciation to all reviewers for their valuable comments.

## Conflict of interest

The authors declare that the research was conducted in the absence of any commercial or financial relationships that could be construed as a potential conflict of interest.

## Publisher’s note

All claims expressed in this article are solely those of the authors and do not necessarily represent those of their affiliated organizations, or those of the publisher, the editors and the reviewers. Any product that may be evaluated in this article, or claim that may be made by its manufacturer, is not guaranteed or endorsed by the publisher.
