# Supplementary material for: Artificial optimization of bamboo Ppmar2 transposase and host factors effects on Ppmar2 transposition in yeast
Source: Front Plant Sci. 2022 Oct 20;13:1004732. doi: 10.3389/fpls.2022.1004732 (PMC9632168; doi:10.3389/fpls.2022.1004732)
Supplement: Supplementary file 1 [file DataSheet_1.docx]

Supplementary Material

Artificial optimization of *Ppmar2* transposase and host factors effects on *Ppmar2* transposition in yeast

Xiaohong Zhou^1^, Jiamin Xie^1^, Chao Xu^1^, Xiuling Cao^1^, Long-Hai Zou^1^, Mingbing Zhou^1^*

^1^ State Key Laboratory of Subtropical Silviculture; Institute of Bamboo, Zhejiang A&F University, Lin’an, Hangzhou 311300, Zhejiang, China

*** Correspondence:**Mingbing Zhou
[zhoumingbing@zafu.edu.cn](mailto:zhoumingbing@zafu.edu.cn)

# Supplementary Figures


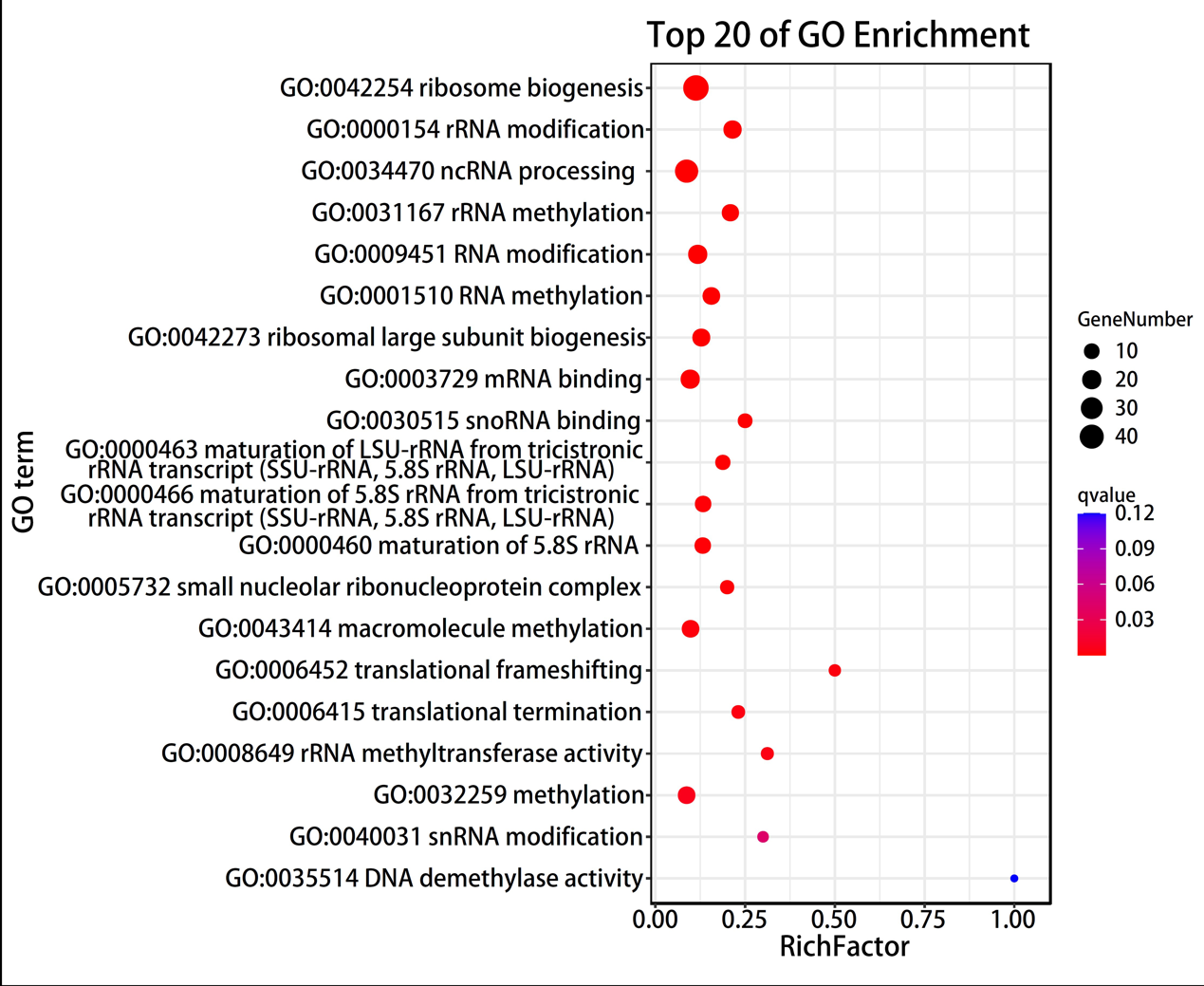


**Supplementary Figure 1.** GO enrichment of the DEGs between *efm1Δ, rtt10Δ* and *HisΔ3* strains. Black ovals indicate the gene number, and q-values indicate the expression level. Rich factor shows the ratio of the DEGs number to the annotated gene number.


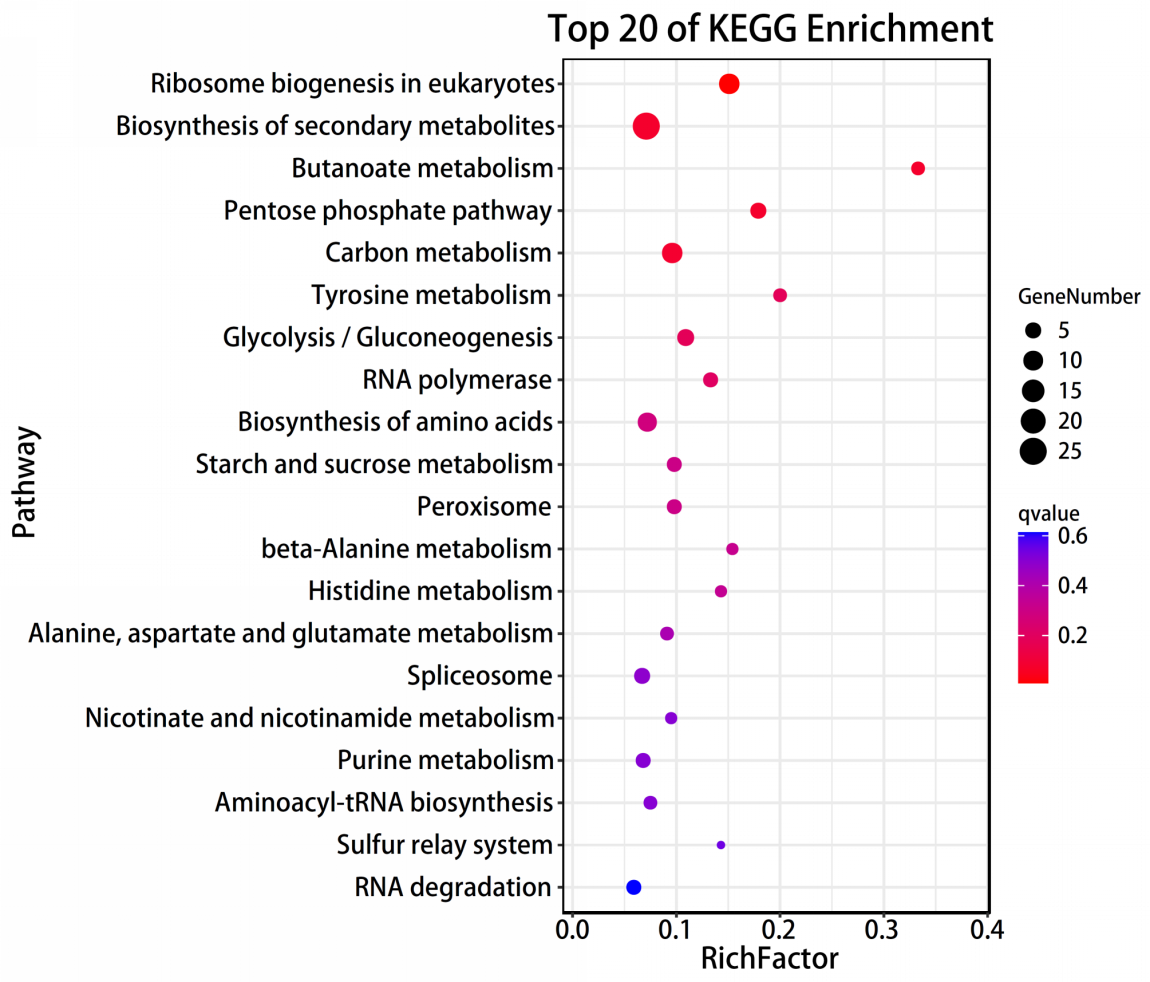


**Supplementary Figure 2.** KEGG enrichment of the DEGs between *efm1Δ, rtt10Δ* and *HisΔ3* strains. Black ovals indicate the gene number, and q-values indicate the expression level. Rich factor shows the ratio of the DEGs number to the annotated gene number.


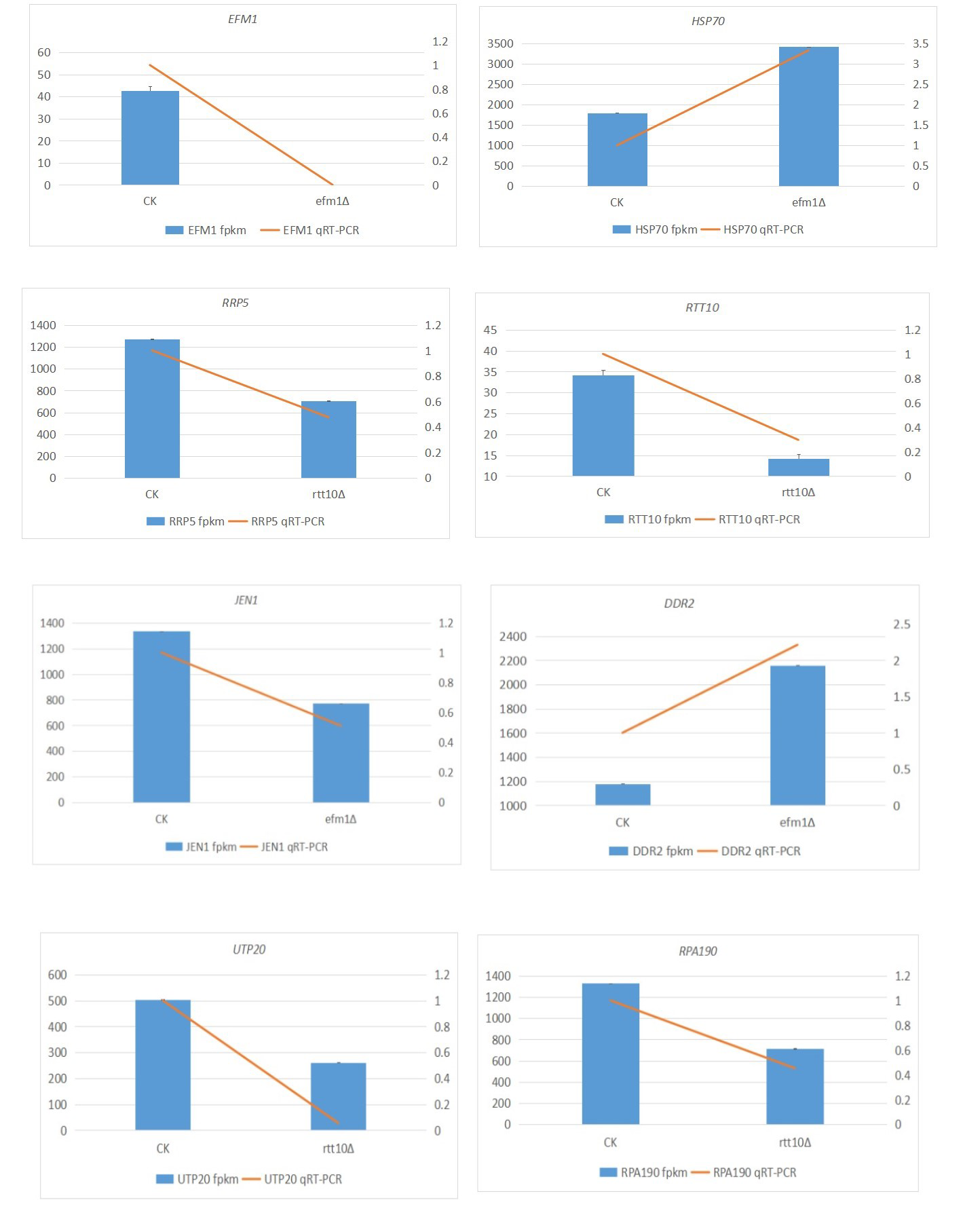


**Supplementary Figure 3.** The expression pattern of *EFM1, RTT10, DDR2, HSP70, JEN1, UTP20, RPA190 and RRP5* displayed by FPKM and qRT-PCR data.

# Supplementary Tables

**Table S1 Primers for the site-directed mutagenesis**

| Primer name | Primer sequence (5’-3’) | notes |
| --- | --- | --- |
| D129A-F | AACGACAATCCGGGCTCTAGCAGGCGCTC | D129A |
| D129A-R | GAGCGCCTGCTAGAGCCCGGATTGTCGTT |  |
| D129R-F | CGAACGACAATCCGGCGTCTAGCAGGCGCTCT | D129R |
| D129R-R | AGAGCGCCTGCTAGACGCCGGATTGTCGTTCG |  |
| G132A-F | AATCCGGGATCTAGCAGCCGCTCTGAATATTTCAA | G132A |
| G132A-R | TTGAAATATTCAGAGCGGCTGCTAGATCCCGGATT |  |
| Q144R-F | CAAAGAGCACATTGTTTAGGCGGATGAAAGAAGGGAAGTTTAG | Q144R |
| Q144R-R | CTAAACTTCCCTTCTTTCATCCGCCTAAACAATGTGCTCTTTG |  |
| M145L-F | GAGCACATTGTTTAGGCAGTTGAAAGAAGGGAAGTTTAG | M145L |
| M145L-R | CTAAACTTCCCTTCTTTCAACTGCCTAAACAATGTGCTC |  |
| A168R-F | TTACATTGACTGAAGATAACAAGAAAAGATGTGTTAAGTTTTGCCTCTCAATG | A168R |
| A168R-R | CATTGAGAGGCAAAACTTAACACATCTTTTCTTGTTATCTTCAGTCAATGTAA |  |
| C169R-F | GACTGAAGATAACAAGAAAGCACGTGTTAAGTTTTGCCTCTCAAT | C169R |
| C169R-R | ATTGAGAGGCAAAACTTAACACGTGCTTTCTTGTTATCTTCAGTC |  |
| L174K-F | AACAAGAAAGCATGTGTTAAGTTTTGCAAGTCAATGCTAGAAAAATTAAGCATGCCG | L174K |
| L174K-R | CGGCATGCTTAATTTTTCTAGCATTGACTTGCAAAACTTAACACATGCTTTCTTGTT |  |
| L235F-F | AAAATTTCATAGAGAAGGTGATGTTCCTCGCAGAAATTGCG | L235F |
| L235F-R | CGCAATTTCTGCGAGGAACATCACCTTCTCTATGAAATTTT |  |
| K243R -F | CGCAGAAATTGCGAGACCTAGATTTGATTGGGATGGAAATG | K243R |
| K243R -R | CATTTCCATCCCAATCAAATCTAGGTCTCGCAATTTCTGCG |  |
| I259F-F | CATTTTCTGGAAAGATAGGCATATTTCCTTTCACTTTCGTAGAGC | I259F |
| I259F-R | GCTCTACGAAAGTGAAAGGAAATATGCCTATCTTTCCAGAAAATG |  |
| L266P- F | CTTTCACTTTCGTAGAGCCAGCAAAGCGAAGTAGTGC | L266P |
| L266P -R | GCACTACTTCGCTTTGCTGGCTCTACGAAAGTGAAAG |  |
| K289A-F | GGCAATGACATCGGTAACCGCGGAAACAAGCCGTGAGTAC | K289A |
| K289A-R | GTACTCACGGCTTGTTTCCGCGGTTACCGATGTCATTGCC |  |
| S292I -F | GACATCGGTAACCAAGGAAACAATCCGTGAGTACCT | S292I |
| S292I -R | AGGTACTCACGGATTGTTTCCTTGGTTACCGATGTC |  |
| V297I -F | ACAAGCCGTGAGTACCTTATAAATAAGGTATTGCCCG | V297I |
| V297I -R | CGGGCAATACCTTATTTATAAGGTACTCACGGCTTGT |  |
| Q306E-F | ATTGCCCGCGATCAAGGAGAAATGGCTAGCGGAGG | Q306E |
| Q306E-R | CCTCCGCTAGCCATTTCTCCTTGATCGCGGGCAAT |  |
| L309P -F | GATCAAGCAAAAATGGCCAGCGGAGGAAGTTGGTA | L309P |
| L309P -R | TACCAACTTCCTCCGCTGGCCATTTTTGCTTGATC |  |
| D333P-F | GGACGCATATTGCAATCAATGATCCCGAGTTTTGTCGTGCG | D333P |
| D333P-R | CGCACGACAAAACTCGGGATCATTGATTGCAATATGCGTCC |  |
| S347R -F | CAGATGGTTTTGACATAAGGTTGATGTGCCAGCCAC | S347R |
| S347R -R | GTGGCTGGCACATCAACCTTATGTCAAAACCATCTG |  |

**Table S2** Distribution of [mutation sites](http://dict.cnki.net/dict_result.aspx?searchword=%e7%aa%81%e5%8f%98%e4%bd%8d%e7%82%b9&tjType=sentence&style=&t=mutation+sites) in *Ppmar2* transposase

| **Mutation sites** | **Location** | **Description** | **Reference** |
| --- | --- | --- | --- |
| D129A* | HTH domain | Corresponding to hyperactive mutation site E90A in *Himar1* | Butler et al., 2006 |
| D129R* | HTH domain | Corresponding to hyperactive mutation site Q91R in *Mos1* | Germon et al., 2009 |
| G132A* | HTH domain | Corresponding to hyperactive mutation site E93A in *Himar1* | Butler et al., 2006 |
| Q144R | HTH domain | Non-conserved site mutated into conserved site | In the study |
| M145L | HTH domain | Non-conserved site mutated into conserved site | In the study |
| A168R* | Linker region | Corresponding to hyperactive mutation site Q131R in *Himar1* | Butler et al., 2006 |
| C169R | Linker region | Non-conserved site mutated into conserved site | In the study |
| L174K* | Linker region | Corresponding to hyperactive mutation site Q137K in *Himar1* | Butler et al., 2006 |
| L235F | DDD domain | Non-conserved site mutated into conserved site | In the study |
| K243R | DDD domain | Non-conserved site mutated into conserved site | In the study |
| I259F | DDD domain | Non-conserved site mutated into conserved site | In the study |
| L266P | DDD domain | Non-conserved site mutated into conserved site | In the study |
| K289A* | DDD domain | Corresponding to hyperactive mutation site T216A in *Mos1* | Germon et al., 2009 |
| S292I | DDD domain | Non-conserved site mutated into conserved site | In the study |
| V297I | DDD domain | Non-conserved site mutated into conserved site | In the study |
| Q306E | DDD domain | Non-conserved site mutated into conserved site | In the study |
| L309P | DDD domain | Non-conserved site mutated into conserved site | In the study |
| D333P | DDD domain | Non-conserved site mutated into conserved site | In the study |
| S347R | DDD domain | Non-conserved site mutated into conserved site | In the study |

*Mutants corresponding to the hyperactive mutation sites in *Mos1* and *Himar1* were marked by red color.

**Table S3 List of the yeast mutants with single-gene deletion**

| **Gene ID** | **ORF** | Gene function |
| --- | --- | --- |
| *SET2** | YJL168C* | histone methylation |
| *LGE1* | YPL055C | histone methylation |
| *JHD1* | YER051W | JmjC domain-containing Histone Demethylase |
| *TMT1** | YER175C* | Trans-aconitate MethylTransferase |
| *EFM1** | YHL039W* | Elongation Factor Methyltransferase |
| *HPM1* | YIL110W | Histidine Protein Methyltransferase |
| *EFM5** | YGR001C* | Elongation Factor Methyltransferase |
| *MGT1** | YDL200C* | O-6-MethylGuanine-DNA methylTransferase |
| *CYC8* | YBR112C | chromatin DNA binding |
| *DPB4* | YDR121W | chromatin DNA binding |
| *RTT106* | YNL206C | Histone chaperone; involved in regulation of chromatin structure in both transcribed and silenced chromosomal regions; affects transcriptional elongation; has a role in regulation of Ty1 transposition; interacts physically and functionally with Chromatin Assembly Factor-1 (CAF-1) |
| *PDR3** | YBL005W* | Retrotransposon TYA Gag and TYB Pol genes; transcribed/translated as one unit; polyprotein is processed to make a nucleocapsid-like protein (Gag), reverse transcriptase (RT), protease (PR), and integrase (IN); similar to retroviral genes |
| *LYS14* | YDR034C | Retrotransposon TYA Gag and TYB Pol genes; transcribed/translated as one unit; polyprotein is processed to make a nucleocapsid-like protein (Gag), reverse transcriptase (RT), protease (PR), and integrase (IN); similar to retroviral genes |
| *GRX3* | YDR098C | Retrotransposon TYA Gag and TYB Pol genes; transcribed/translated as one unit; polyprotein is processed to make a nucleocapsid-like protein (Gag), reverse transcriptase (RT), protease (PR), and integrase (IN); similar to retroviral genes |
| *YDR210W* | YDR210W | Retrotransposon TYA Gag and TYB Pol genes; transcribed/translated as one unit; polyprotein is processed to make a nucleocapsid-like protein (Gag), reverse transcriptase (RT), protease (PR), and integrase (IN); similar to retroviral genes |
| *EXG2** | YDR261C* | Retrotransposon TYA Gag and TYB Pol genes; Gag processing produces capsid proteins, Pol is cleaved to produce protease, reverse transcriptase and integrase activities; in YDRCTy1-3 TYB is mutant and probably non-functional |
| *OMS1* | YDR316W | Retrotransposon TYA Gag and TYB Pol genes; transcribed/translated as one unit; polyprotein is processed to make a nucleocapsid-like protein (Gag), reverse transcriptase (RT), protease (PR), and integrase (IN); similar to retroviral genes |
| *RTS3* | YGR161C | Retrotransposon TYA Gag and TYB Pol genes; transcribed/translated as one unit; polyprotein is processed to make a nucleocapsid-like protein (Gag), reverse transcriptase (RT), protease (PR), and integrase (IN); similar to retroviral genes |
| *MLH2** | YLR035C* | Retrotransposon TYA Gag and TYB Pol genes; polyprotein is processed to make a nucleocapsid-like protein (Gag), reverse transcriptase (RT), protease (PR), and integrase (IN); YLR035C-A is part of a mutant retrotransposon |
| *VIP1* | YLR410W | Retrotransposon TYA Gag and TYB Pol genes; transcribed/translated as one unit; polyprotein is processed to make a nucleocapsid-like protein (Gag), reverse transcriptase (RT), protease (PR), and integrase (IN); similar to retroviral genes |
| *VAC7* | YNL054W | TyB Gag-Pol protein; proteolytically processed to make the Gag, reverse transcriptase (RT), protease (PR), and integrase (IN) proteins that are required for retrotransposition |
| *MRPL10** | YNL284C* | Retrotransposon TYA Gag and TYB Pol genes; transcribed/translated as one unit; polyprotein is processed to make a nucleocapsid-like protein (Gag), reverse transcriptase (RT), protease (PR), and integrase (IN); similar to retroviral genes |
| *ITR2** | YOL103W* | Retrotransposon TYA Gag and TYB Pol genes; transcribed/translated as one unit; polyprotein is processed to make a nucleocapsid-like protein (Gag), reverse transcriptase (RT), protease (PR), and integrase (IN); similar to retroviral genes |
| *LSC1* | YOR142W | Retrotransposon TYA Gag and TYB Pol genes; transcribed/translated as one unit; polyprotein is processed to make a nucleocapsid-like protein (Gag), reverse transcriptase (RT), protease (PR), and integrase (IN); similar to retroviral genes |
| *THI72* | YOR192C | Retrotransposon TYA Gag and TYB Pol genes; transcribed/translated as one unit; polyprotein is processed to make a nucleocapsid-like protein (Gag), reverse transcriptase (RT), protease (PR), and integrase (IN); similar to retroviral genes |
| *YPL257W* | YPL257W | Retrotransposon TYA Gag and TYB Pol genes; transcribed/translated as one unit; polyprotein is processed to make a nucleocapsid-like protein (Gag), reverse transcriptase (RT), protease (PR), and integrase (IN); similar to retroviral genes |
| *CUR1** | YPR158W* | Retrotransposon TYA Gag and TYB Pol genes; transcribed/translated as one unit; polyprotein is processed to make a nucleocapsid-like protein (Gag), reverse transcriptase (RT), protease (PR), and integrase (IN); similar to retroviral genes |
| *RTT107* | YHR154W | Regulator of Ty1 Transposition |
| *RTT103* | YDR289C | Regulator of Ty1 Transposition |
| *RTT102** | YGR275W* | Regulator of Ty1 Transposition |
| *RTT101* | YJL047C | Regulator of Ty1 Transposition |
| *RTT109* | YLL002W | Regulator of Ty1 Transposition |
| *HRT3* | YLR097C | High level expression Reduces Ty3 transposition |
| *RTT10** | YPL183C* | Regulator of Ty1 Transposition |
| *CHD1** | YER164W* | Chromatin organization modifier, Helicase, and DNA-binding domains |
| *DPB4* | YDR121W | during DNA replication, nucleosome mobilization, and heterochromatin organization that contributes to chromatin |
| *PCC1* | YKR095W-A | Polarized growth Chromatin-associated Controller |
| *CYC8* | YBR112C | General transcriptional co-repressor; acts together with Tup1p; also acts as part of a transcriptional co-activator complex that recruits the SWI/SNF and SAGA complexes to promoters; can form the prion [OCT+] |
| *GON7* | YJL184W | Component of the EKC/KEOPS protein complex; EKC/KEOPS complex is required for t6A tRNA modification and telomeric TG1-3 recombination |
| *KAE1* | YKR038C | Subunit of EKC/KEOPS complex capable of chromatin binding; contributes to telomere maintenance via recombination |
| *RED1* | YLR263W | Chromatin-binding structural molecule of lateral element proteinaceous core found between sister chromatids |
| *TGS1* | YPL157W | Trimethyl guanosine synthase, conserved nucleolar methyl transferase; converts the m(7)G cap structure |

*Mutants where *Ppmar2* transposition activity increased more than 2 times were marked by red color and reduced to 1/2 were marked by green color.
